# Molecular characterization and expression analysis of B-cell lymphoma-2 in *Trachinotus ovatus* and its role in apoptotic process

**DOI:** 10.3389/fimmu.2023.1129800

**Published:** 2023-03-16

**Authors:** Zhenjie Cao, Xin Yang, Tao Li, Zhiru Liu, Pengfei Li, Yongcan Zhou, Yun Sun

**Affiliations:** ^1^ State Key Laboratory of Marine Resource Utilization in South China Sea, Hainan University, Haikou, China; ^2^ Collaborative Innovation Center of Marine Science and Technology, Hainan University, Haikou, China; ^3^ Guangxi Key Laboratory of Marine Natural Products and Combinatorial Biosynthesis Chemistry, Guangxi Beibu Gulf Marine Research Center, Guangxi Academy of Sciences, Nanning, Nanning, China

**Keywords:** *Trachinotus ovatus*, Bcl-2, mitochondria, apoptosis, NF-κB

## Abstract

**Introduction:**

B-cell lymphoma-2 (Bcl-2) is the first identified member of the Bcl-2 family that performs an anti-apoptotic function in mammals. However, its role in teleosts is not fully understood. In this study, Bcl-2 of *Trachinotus ovatus* (TroBcl2) was cloned, and its role in apoptosis was investigated.

**Methods:**

In this study, Bcl-2 of *Trachinotus ovatus* (TroBcl2) was cloned by PCR. Quantitative real-time PCR (qRT-PCR) was used to detect its mRNA expression level in healthy condition and after LPS stimulation. Subcellular localization was performed by transfecting the pTroBcl2-N3 plasmid into golden pompano snout (GPS) cells and observed under an inverted fluorescence microscope DMi8 and further verified by immunoblotting. *In vivo* overexpression and RNAi knockdown method were performed to evaluate the role of TroBcl2 in apoptosis. The anti-apoptotic activity of TroBcl2 was detected by flow cytometry. The effect of TroBcl2 on the mitochondrial membrane potential (MMP) was measured by an enhanced mitochondrial membrane potential assay kit with JC-1. The terminal deoxynucleotidyl transferase-mediated dUTP nick end labeling (TUNEL) method was performed to evaluate the role of TroBcl2 in the DNA fragmentation. Immunoblotting was used to verify whether TroBcl2 inhibits the release of cytochrome c from mitochondria into the cytoplasm. The Caspase 3 and Caspase 9 Activity Assay Kits were used to investigate the effect of TroBcl2 on caspase 3 and caspase 9 activities. The effects of TroBcl2 on the expression of apoptosis-related and nuclear factor- κB (NF-κB) signaling pathway-related genes *in vitro* were evaluated by qRT-PCR and Enzyme linked immunosorbent assay (ELISA). Luciferase reporter assay was used to evaluate the activity in NF-κB signaling pathway.

**Results and discussion:**

The full-length coding sequence of TroBcl2 contains 687 bp and encodes a protein containing 228 amino acids. Four conserved Bcl-2 homology (BH) domains and one invariant “NWGR” motif located in BH1 were identified in TroBcl2. In healthy *T. ovatus*, TroBcl2 was widely distributed in the eleven tested tissues, and higher expression levels were found in immune-related tissues, such as spleen and head kidney tissues. After stimulation with lipopolysaccharide (LPS), the expression of TroBcl2 in the head kidney, spleen, and liver was significantly upregulated. In addition, subcellular localization analysis revealed that TroBcl2 was localized in both the cytoplasm and nucleus. Functional experiments showed that TroBcl2 inhibited apoptosis, possibly by reducing mitochondrial membrane potential loss, decreasing DNA fragmentation, preventing cytochrome c release into cytoplasm, and reducing the caspase 3 and caspase 9 activations. Moreover, upon LPS stimulation, overexpression of TroBcl2 suppressed the activation of several apoptosis-related genes, such as *BOK, caspase-9, caspase-7, caspase-3, cytochrome c*, and *p53*. Furthermore, knockdown of TroBcl2 significantly increased the expression of those apoptosis-related genes. In addition, TroBcl2 overexpression or knockdown induced or inhibited, respectively, the transcription of NF-κB and regulated the expression of genes (such as *NF-κB1* and *c-Rel*) in the NF-κB signaling pathway as well as the expression of the downstream inflammatory cytokine *IL-1β*. Overall, our study suggested that TroBcl2 performs its conserved anti-apoptotic function via the mitochondrial pathway and may serve as an anti-apoptotic regulator in *T. ovatus*.

## Introduction

Apoptosis is an indispensable physiological process for normal biological activities of cells and is characterized by the interaction of a group of specific proteins to transmit programmed death-inducing signals to eliminate senescent, redundant or dysfunctional cells (1–3). Apoptosis is widely observed in multicellular organisms and plays a key role in the regulation of embryonic development, autophagy, homeostasis, and the host immune system (4, 5). Recent studies in mammals have revealed that apoptosis is mainly regulated by the following signaling pathways: the death receptor pathway (or extrinsic pathway) triggered by death receptors on the cell surface; the mitochondrial pathway (or intrinsic pathway) initiated by a number of stress conditions, chemotherapeutic agents and drugs; the endoplasmic reticulum stress (ERS) pathway (6–8). In vertebrate cells, the mitochondrial pathway is one of the two major apoptosis pathways (extrinsic and intrinsic pathways), with mitochondrial outer membrane permeabilization (MOMP) as the key event (9). It is clear that a change in MOMP causes apoptosis through the release of pro-apoptotic substances (e.g., cytochrome c, apoptosis inducing factor (AIF), high-temperature requirement A2 (HtrA2), and second mitochondria-derived activator of caspases (Smac)) into the cytoplasm, which in turn activates the initiator caspase (caspase 9) and executioner caspases (caspases 3, 6, and 7) and therefore results in apoptosis (10).

Accumulating evidence has shown that B-cell lymphoma-2 (Bcl-2) family members can directly control MOMP by regulating the membrane potential (11). Numerous studies have shown that Bcl-2 family members are the core regulators of intrinsic apoptosis. They control cellular apoptosis and survival, and are deeply and widely studied molecules in apoptosis (12–15). A characteristic feature of Bcl-2 family members is the presence of Bcl-2 homology (BH) domains, which provide the foundation of the family members’ interactions with each other. According to their structure and function, the Bcl-2 family is subdivided into three subfamilies: anti-apoptotic proteins (such as Bcl-2, Mcl-1, Bcl-w, Bcl-x, and A1), multidomain pro-apoptotic proteins (such as Bok, Bax, and Bak), and pro-apoptotic BH3-only proteins (such as Bad, Bik, Bid, Bim, Puma, Beciln-1, and Noxa) (16). Whether a cell lives or dies mainly depends on the dynamic balance between pro-apoptotic and anti-apoptotic proteins, and when this balance is disrupted, many diseases (such as autoimmune diseases and cancer) ensue (17, 18). In unstimulated cells, interactions between anti-apoptotic and multidomain pro-apoptotic proteins prevent the oligomerization of pro-apoptotic proteins and initiate the subsequent apoptotic program. Upon receipt of apoptotic signals, the activities of anti-apoptotic proteins are inhibited by pro-apoptotic BH3-only proteins, thereby indirectly activating multidomain pro-apoptotic proteins, freeing them to initiate apoptosis (19).

Bcl-2 was the first identified member of the Bcl-2 family; it belongs to the anti-apoptotic subfamily and functions by inhibiting apoptosis or promoting cell survival (20, 21). In mammals, much evidence has confirmed that Bcl-2 displays its ability in the apoptotic process. Early studies reported that overexpression of Bcl-2 in myeloid cells, intestinal epithelial cells, or T/B cells can reduce apoptosis in these cell types and promote the survival of septic mice (22–24). Irradiation- and dexamethasone-induced cell death can be suppressed by overexpression of Bcl-2 (25–27). In mice subjected to cecal ligation and puncture (CLP), rhBcl-2 treatment significantly decreased the numbers of apoptotic neutrophils in the intestine and heart and protected mice from sepsis (28). In addition, apoptosis induced by docosahexaenoic acid (DHA; 22:6*n*-3) and butyrate in colon cells from young adult mice was blocked by overexpression of Bcl-2 (29). As reported, Bcl-2 performs its anti-apoptotic function by forming heterodimers with pro-apoptotic proteins (such as Bax), thereby preventing their pro-apoptotic effects and inhibiting cytochrome c release and the subsequent caspase cascade in cells (30, 31). Additionally, Bcl-2 can maintain the MOMP by regulating the Ca^2+^ concentration and antioxidant effects, thus ensuring cell survival (31).

To date, several Bcl-2 homologous sequences have been cloned and characterized in teleosts, such as *Danio rerio*, *Channa striatus*, *Takifugu obscurus*, *Ictalurus punctatus*, and *Epinephelus coioides* (2, 4, 32–34). The anti-apoptotic function of fish Bcl-2 seems to be conserved compared with that of mammalian Bcl-2. In orange-spotted grouper, overexpression of EcBcl-2 can inhibit lipopolysaccharide (LPS)-induced apoptosis of HeLa cells (4). Zebrafish Bcl-2 can block irradiation- and dexamethasone-induced apoptotic responses in lymphoid cells, increase the numbers of thymocytes and B cells in the kidney, and alter thymocyte homeostasis (35). However, the understanding of the roles of fish Bcl-2 in apoptosis is still limited, and nothing is known specifically about Bcl-2 of Trachinotus ovatus, despite the economic importance of this farmed marine fish.

In the current study, we cloned and characterized a new Bcl-2 gene from *T. ovatus* (named TroBcl2). The tissue distribution of the TroBcl2 transcript and its expression profiles upon LPS stimulation were investigated. Then, the intercellular localization of TroBcl2 was explored in golden pompano snout (GPS) cells. Moreover, the function and mechanism of TroBcl2 in apoptosis were examined in GPS cells. These findings reveal the functions of TroBcl2 in *T. ovatus* and enrich our understanding of the regulatory mechanism of fish Bcl-2.

## Materials and methods

### Ethics statement

Experiments involving live animals were conducted in accordance with the “Regulations for the Administration of Affairs Concerning Experimental Animals” promulgated by the State Science and Technology Commission of Hainan Province. The study was approved by the Animal Care and Use Committee of the Hainan University.

### Fish and sampling

*Trachinotus ovatus* (average weight 15.50 ± 0.32 g) were obtained from a commercial farm in Hainan Province, China. Prior to experiments, the fish were cultured in aerated seawater to adapt to the laboratory environment (27 ± 1°C). As reported previously, several fish were randomly chosen to ensure that there was no pathogenic infection before the start of the experiment (36). Before anatomical sampling, fish were anesthetized using MS-222.

### Gene cloning, sequence and phylogenetic analysis of TroBcl2

The complete coding sequence (CDs) of *TroBcl2* were obtained from the *T. ovatus* transcriptome data (GenBank accession no: PRJNA505850) and was then amplified by PCR with the primer pair of TroBcl2-F and TroBcl2-R (**Table S1**). The molecular weight and isoelectric point (PI) were predicted by the ExPASy tool (http://www.expasy.org/tools). The conserved structures of the TroBcl2 protein sequence were analyzed by SMART (http://smart.embl-heidelberg.de) and InterProScan (https://www.ebi.ac.uk/interpro/search/sequence/). The multiple sequence alignment and sequence similarity of Bcl-2 were analyzed by DNAMAN software. A phylogenetic tree of Bcl-2 was constructed by MEGA 6.0 with the neighbor-joining (NJ) method, and the number of bootstrap replications was set to 1000.

### Tissue distribution, LPS challenge and quantitative real-time PCR analysis

To investigate the tissue distribution of *TroBcl2* in *T. ovatus*, various tissues (blood, head kidney, gill, intestine, stomach, liver, spleen, muscle, skin, heart, and brain) were taken from 15 healthy fish under sterile conditions and the same tissue from five consecutive fish was mixed together as a parallel. All removed tissues were immediately stored in liquid nitrogen for further use.

To examine the changes in *TroBcl2* expression under LPS stimulation, 150 fish were randomly divided into two equal groups. In the experimental group, the fish were intraperitoneally injected with 100 μL of LPS (20 μg/ml). In the control group, the fish were intraperitoneally injected with 100 μL of PBS. At 6, 12, 24, 48, and 72 h after infection, the liver, spleen, and head kidney from 15 fish of each group were removed and pooled as three replicates for each time point. All removed tissues were immediately preserved in liquid nitrogen for subsequent RNA extraction.

The tissues stored as described above were processed for RNA extraction, and total RNA was isolated according to the instructions of the Easystep^®^ Super Total RNA Extraction Kit (Promega, Madison, WI, USA). Then, synthesis of first-strand cDNA was performed according to the protocols of the Easystep^®^ RT Master Mix Kit (Promega, Madison, WI, USA) with the total RNA as the template.

qRT-PCR was executed in the Quant-Studio^™^ 6 Real-Time PCR System (ABI, Vernon, CA, USA) using the Easystep^®^ qPCR Master Mix Kit (Promega, Madison, USA) with the synthesized cDNA serving as a template. The mRNA expression pattern and changes in the temporal expression of TroBcl2 in response to LPS treatment were determined by qRT-PCR with the primers TroBcl2-RT-F and TroBcl2-RT-R (**Table S1**). The reference gene was *beta-2-microglobulin* (*B2M*), which was confirmed in our previous study, and its expression level was measured with the primers B2M-RT-F and B2M-RT-R listed in **Table S1** (37). The 2^−ΔΔCt^ method was used to calculate the expression level of TroBcl2 relative to the control. There were three independent replicates for all experiments.

### Plasmid construction and RNA interference

To construct the recombinant eukaryotic TroBcl2 expression plasmid (named pTroBcl2), the CDs of TroBcl2 was inserted into the *EcoR* V restriction site in the pCN3 vector, as reported in our previous study (38). To investigate the subcellular localization of TroBcl2, a recombinant eukaryotic expression plasmid (named pTroBcl2-N3) that can simultaneously express a green fluorescent protein (GFP) and the TroBcl2 protein was constructed. Briefly, the CDs of TroBcl2 was ligated into the *EcoR* V restriction site in the pEGFPX-N3 vector, which was modified from the original vector of pEGFP-N3 (BD Biosciences, Clontech) (38). All successfully constructed recombinant plasmids were confirmed by sequencing.

Small interfering RNA (siRNA)-mediated knockdown of TroBcl2 (siTroBcl2) was performed by the T7 RiboMAX™ Express RNAi System (Promega, Madison, WI, USA) following the manufacturer’s protocol. The primers used for the synthesis of siTroBcl2 and the control siRNA (siTroBcl2-C) are listed in **Table S1**.

### Cell culture and subcellular localization

To better explore the biological functions of TroBcl2, golden pompano snout (GPS) cells line was used in subsequent experiments (such as subcellular localization, flow cytometry, mitochondrial membrane potential, TUNEL assay, and so on) (39). Cells were seeded in a 6-well plate (Corning Inc., New York, USA) and cultured in Leibovitz’s L-15 medium (containing 10% fetal bovine serum (FBS; Gibco), 0.5% of 1 M N-2-hydroxyethylpiperazine-N’-2 ethanesulfonic acid (HEPES) (Thermo, USA), 1% sodium chloride, and 4% antibiotics) at 26°C to approximately 70% confluence. Then, 1 μg pTroBcl2-N3 or 1 μg pEGFPX-N3 empty plasmid (control) was transfected into GPS cells using Lipofectamine 3000 (Thermo Fisher Scientific Lipofectamine, Shanghai, CHN) following the manufacturer’s protocol. After transfection for 48 h, the transfected cells were washed three times with PBS, fixed with 4% paraformaldehyde for 10 min, and stained with 1 μg/ml 4’,6-diamidino-2-phenylindole (DAPI) for 20 min. After staining, the cells were washed three times with PBS and observed under an inverted fluorescence microscope DMi8 (Leica Microscopes, Wetzlar, GER). The experiment was performed in triplicate.

### Flow cytometry assay

To evaluate the effect of TroBcl2 overexpression on apoptosis, 1 × 10^6^ GPS cells were seeded in a 6-well plate and transfected with pTroBcl2 or pCN3 *via* the transfection procedure described above. Furthermore, to detect the effect of TroBcl2 knockdown on apoptosis, GPS cells were transfected with siTroBcl2 or siTroBcl2-C using a *TransIntro* ^®^ EL Transfection Reagent kit (Transgen, Beijing, China) according to the manual. Before LPS stimulation, the overexpression and knockdown efficiencies of TroBcl2 was confirmed by measuring the mRNA and protein expression levels of TroBcl2 using qRT-PCR with the primers TroBcl2-RT-F and TroBcl2-RT-R and western blotting. After that, 2 μg/ml LPS was added to stimulate cells after 24 h post-transfection. Non-transfected cells treated with LPS were as the control. After treatment for 24 h, the cells were washed with PBS and then the apoptosis was analyzed with an Annexin V FITC Apoptosis Detection Kit I (BD Pharmingen™, San Jose, CA, USA) in a Guava easyCyte™ Flow Cytometer (EMD Millipore Corp., Billerica, USA). Data were processed by guavaSoft 3.1.1. There were three independent replicates for the experiment.

### Measurement of the mitochondrial membrane potential

To measure the effect of TroBcl2 on the MMP, GPS cells were transfected with pTroBcl2 (or pCN3), and siTroBcl2 (or siTroBcl2-C) according to the transfection procedure described above. After transfection for 24 h, 2 μg/ml LPS was used to stimulate transfected cells for 24 h, as previously reported (4, 40). After that, the MMP changes in transfected GPS cells were measured by an enhanced mitochondrial membrane potential assay kit with JC-1 (Beyotime, Shanghai, China) in accordance with the instructions. Red and green fluorescence were visualized with an inverted fluorescence microscope (DMi8, Leica Microscopes, Wetzlar, GER). The experiment was performed three times.

### TUNEL assay

To continue to evaluate the role of TroBcl2 in the process of apoptosis, the terminal deoxynucleotidyl transferase-mediated dUTP nick end labeling (TUNEL) method was performed. Briefly, the pTroBcl2 plasmid or siTroBcl2 RNA was transfected into GPS cells as described above, and the pCN3 plasmid or siTroBcl2-C RNA was used as the control. Twenty-four hours after transfection, the cells were stimulated with different concentrations of LPS (0, 2, and 4 μg/ml). After stimulation for 24 h, the cells were washed with PBS once and fixed with 4% paraformaldehyde for 30 min. Afterwards, the cells were washed with PBS once again and incubated with PBS containing 0.3% Triton X-100 for 5 min at room temperature. Subsequently, the cells were washed with PBS twice and incubated with the TUNEL reaction mixture at 37°C in the dark for 60 min. Then, after washing with PBS three times, the cells were sealed with anti-fluorescence quenching buffer and visualized with an inverted fluorescence microscope (DMi8, Leica Microscopes, Wetzlar, GER). The experiment was performed three times.

### Cytochrome c release assay

To evaluate whether TroBcl2 inhibits the release of cytochrome c from mitochondria into the cytoplasm, we examined changes in cytochrome c protein levels in mitochondria-free cytoplasm after TroBcl2 overexpression and knockdown in GPS cells. Briefly, GPS cells were transfected with pTroBcl2 (or pCN3), and siTroBcl2 (or siTroBcl2-C) according to the transfection procedure described above. 24 h after transfection, cells were stimulated by 2 μg/ml LPS. Non-transfected cells treated with LPS were as the control. Twenty-four hours after LPS stimulation, proteins were isolated from of mitochondria-free cytoplasm using a cell mitochondrial isolation kit (Beyotime, Shanghai, China) and then analyzed using western blotting with a mouse anti-TroCyt c polyclonal antibody (stored in our laboratory, 1:1,000 dilution) as the primary antibody and HRP-conjugated goat anti-mouse IgG (1:2,000 dilution) as the secondary antibody. An anti-β-actin mouse monoclonal antibody (Bioss, Beijing, China) was used as an internal reference. Immunoreactions were detected with a supersensitive ECL substrate (Biosharp, Anhui, China).

### Caspases activity assay

To investigate the role of TroBcl2 on caspases activation during LPS stimulation, the activities of caspase 3 and caspase 9 were examined by the Caspase 3 and Caspase 9 Activity Assay Kits (Beyotime, Shanghai, China) according to the manuals. GPS cells were transfected with pTroBcl2 (or pCN3), and siTroBcl2 (or siTroBcl2-C) according to the transfection procedure described above. Then, 24 h after transfection, cells were stimulated by 2 μg/ml LPS. Non-transfected cells treated with LPS were as the control. Twenty-four hours after LPS stimulation, cells were digested and collected by centrifugation with 600 g at 4°C for 5 min. Then, the supernatant was removed and the precipitate cells was cleaned once with PBS. After that, cells were lysed with lysate buffer in ice bath for 15 min. Then cells were centrifuged at 20,000 g for 15 min at 4°C. The supernatant was used for detecting the caspase activity. The Ac-DEVD-*p*NA and Ac-LEHD-*p*NA were the substrates for caspase 3 and caspase 9, respectively. The absorbance at 405 nm was measured using a microplate reader of multi-wavelength (Thermo Fisher Scientifi, BioTek, USA). There were three independent replicates for the experiment.

### Effect of TroBcl2 on the expression of apoptosis-related and NF-κB signaling pathway-related genes *in vitro*

#### TroBcl2 overexpression

GPS cells were transfected with pTroBcl2 or pCN3 as described above. At 24 h post-transfection, cells were stimulated with 2 μg/ml LPS. The control group was non-transfected cells treated with LPS. After stimulation by LPS for 24 h, cells were collected for total RNA extraction. Next, the mRNA expression of apoptosis-related genes (including *Bcl2 ovarian killer* (*BOK*), *caspase 3*, *caspase 7*, *caspase 9*, *p53*, and *cytochrome c*) and NF-κB signaling pathway-related genes (*NF-κB1*, *c-Rel*, and *IL-1β*) was evaluated by qRT-PCR after LPS treatment. The primers used for amplification of these genes are listed in **Table S1**. The experiment was performed three times.

#### TroBcl2 knockdown

GPS cells were transfected with siTroBcl2 or siTroBcl2-C as described above. At 24 h post-transfection, cells were stimulated by 2 μg/ml LPS. The control group was non-transfected cells treated with LPS. Likewise, total RNA was extracted from cells stimulated with 2 μg/ml LPS for 24 h. The mRNA expression levels of the apoptosis-related genes and NF-κB signaling pathway-related genes mentioned above were measured by qRT-PCR as described above. The experiment was performed three times.

### Luciferase reporter assay

GPS cells were co-transfected with NF-κB-pGL4.32 (luc2P/NF-kB-RE/Hygro, Promega, USA), pRL-CMV (a control vector), pTroBcl2 or pCN3 or siTroBcl2 or siTroBcl2-C. After 24 h of transfection, cells were treated with 2 μg/ml LPS for 24 h. The control group was non-transfected cells treated with LPS. Then, firefly luciferase activity was measured according to the manufacturer’s protocol for the Dual-Luciferase Reporter Assay Kit (Vazyme, China). The experiment was performed three times.

### Enzyme linked immunosorbent assay

To further verify the effect of TroBcl2 on the expression of BOK and p53 protein, ELISA assay was performed using BOK (fish Bcl-2 associated ovarian Killer protein) ELISA kit and fish tumor protein p53 (TP53) ELISA kit (Zeye Biotechnology, Shanghai, China), respectively. Briefly, GPS cells were transfected with pTroBcl2 or pCN3, siTroBcl2 or siTroBcl2-C as described above. At 24 h post-transfection, cells were stimulated with 2 μg/ml LPS. The control group was non-transfected cells treated with LPS. After stimulation by LPS for 24 h, cells were collected and lysed by repetitive freeze-thawing. Then, the contents of BOK and p53 protein in cells were detected according to the manuals of ELISA kits.

### Western blot analysis

To evaluate the TroBcl2 protein overexpression and knockdown efficiencies, total protein from GPS cells transfected with pTroBcl2 or siTroBcl2 was extracted and analyzed using western blotting with a mouse anti-His (monoclonal, 1:1,000 dilution) primary antibody and HRP-conjugated goat anti-mouse IgG (1:2,000 dilution) as the secondary antibody. An anti-β-actin mouse monoclonal antibody (Bioss, Beijing, China) was used as an internal reference. Immunoreactions were detected with a supersensitive ECL substrate (Biosharp, Anhui, China).

To detect the subcellular localization of TroBcl2, GPS cells were transfected with pTroBcl2-N3 or pEGFPX-N3. The Nuclear and Cytoplasmic Extraction Reagents Kit (Beyotime, Beijing, China) was used to separately extract the nuclear and cytoplasmic proteins. The primary antibodies were mouse anti-TroBcl2 polyclonal antibody (1:2,000 dilution), and the secondary antibody was HRP-conjugated goat anti-mouse IgG (1:2,000 dilution, Bioss, Beijing, China). Anti-Tubulin and anti-Histone H3 (Bioss, Beijing, China) were used as the nucleus and cytoplasm internal reference, respectively.

### Statistical analysis

All data were processed with GraphPad Prism software (version 8, La Jolla CA, USA). Data are presented as the mean ± standard deviation (SD). The significance of differences was determined by one-way analysis of variance (ANOVA) with thresholds of *P* < 0.05 (*) and *P* < 0.01 (**).

## Results

### Sequence and structural analyses of TroBcl2

**Figure 1:**
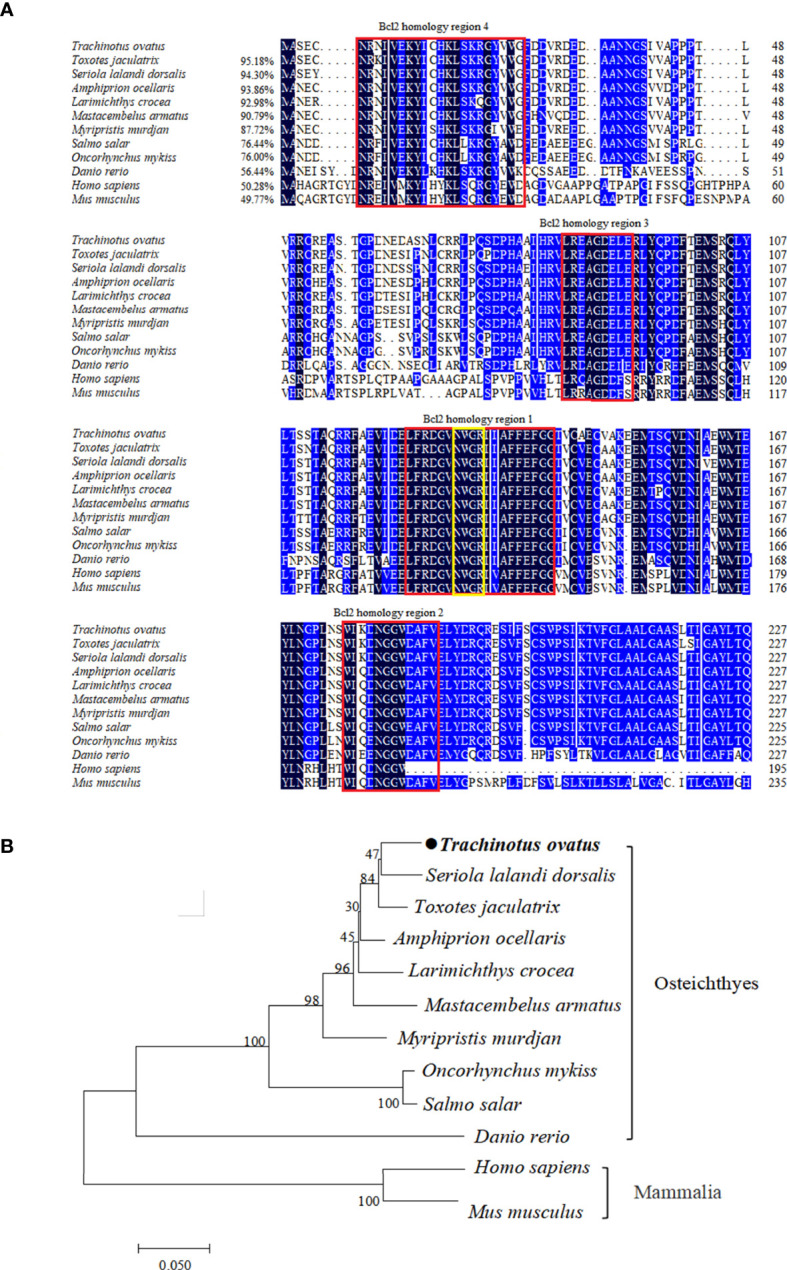
Multiple alignment and phylogenetic analysis of TroBcl2 amino acid sequence with Bcl-2 from other species. Multiple alignment of TroBcl2 with Bcl-2 from other species was performed **(A)**. The sequence similarities between TroBcl2 and other species Bcl-2 were shown in parentheses. The black background color represents fully conserved amino acids, while blue background color represents amino acids with ≥75% identical among the aligned sequences. Four Bcl-2 homology regions were shown in red boxes. The conserved “NWGR” motif was marked with yellow box. GenBank accession numbers of Bcl-2 amino acid sequences used for homologous alignment can be found in **Table S2**. The phylogenetic relationship between TroBcl2 of *Trachinotus ovatus* with Bcl-2 from other species was analyzed by MEGA6 using the neighbor-joining method with 1000 bootstrap replications **(B)**. GenBank accession numbers of Bcl-2 amino acid sequences used for phylogenetic analysis can be found in **Table S2**.

The *TroBcl2* CDs contains 687 bp and encodes a protein with 228 amino acids (aa) (GenBank accession number OP784574). The predicted molecular weight and pI of the TroBcl2 protein are 25.75 kDa and 4.86, respectively. By analysis of protein domains, four conserved Bcl-2 homology (BH) regions were predicted: the BH4 motif (aa 6-26), BH3 motif (aa 84-92), BH1 motif (aa 124-142), and BH2 motif (aa 176-187) (**Figure 1A**). Moreover, the highly conserved invariant “NWGR” motif was identified in BH1. Multiple alignment of Bcl-2 aa sequences showed that TroBcl2 shares high homology with Bcl-2 of other vertebrates, such as *Toxotes jaculatrix* (95.18%), *Larimichthys crocea* (92.98%), *Salmo salar* (76.44%), *Danio rerio* (56.44%), and *Homo sapiens* (50.28%) (**Figure 1A**; **Table S2**). TroBcl2 exhibits the lowest identity (49.77%) with Bcl-2 of *Mus musculus*. A phylogenetic tree based on the aa sequences of Bcl-2 from multiple species revealed that TroBcl2 is first clustered with Bcl-2 from other Osteichthyes and then with groups with mammalian Bcl-2 (**Figure 1B**), consistent with the concept of traditional taxonomy.

### Tissue distribution and changes in the mRNA expression of TroBcl2 under LPS stimulation

**Figure 2:**
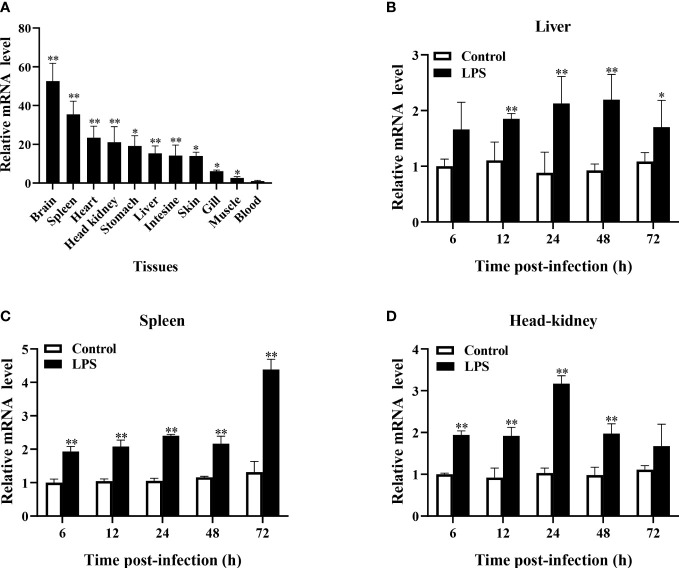
Expression profiles of *TroBcl2* mRNA in healthy *Trachinotus ovatus* and after LPS stimulation. The distribution of *TroBcl2* mRNA in various tissues of healthy *T. ovatus* was detected by qRT-PCR **(A)**. The relative mRNA expression level of *TroBcl2* was calculated, while the lowest mRNA transcript level in the blood was set to 1. The mRNA expression profiles of *TroBcl2* were investigated in the liver **(B)**, spleen **(C)**, and head kidney **(D)** at various time points post LPS challenge were investigated by qRT-PCR. The internal reference gene in both experiments was *B2M*. Vertical bars represent means ± SD. **P < 0.01, *P < 0.05.

qRT-PCR analysis revealed that *TroBcl2* was extensively distributed in all tested tissues (blood, head kidney, gill, intestine, stomach, liver, spleen, muscle, skin, heart, and brain) to varying degrees. The lowest expression level of *TroBcl2* was observed in blood (set to 1). The highest expression level of *TroBcl2* was observed in the brain (52.65-fold), followed by in the spleen (35.46-fold), heart (23.38-fold), head kidney (21.07-fold), stomach (19.14-fold), liver (15.36-fold), intestine (14.23-fold), skin (14.01-fold), gill (6.15-fold), and muscle (2.65-fold) (**Figure 2A**).

In response to LPS stimulation, *TroBcl2* expression in the liver was markedly increased after 12, 24, 48, and 72 h in comparison with that in the control group, peaking at 48 h (2.20-fold) (*P* < 0.01) (**Figure 2B**). Moreover, the mRNA expression level of *TroBcl2* in the spleen was significantly increased at time points from 6 h to 72 h after LPS challenge compared with that in the control group, with the highest expression at 72 h post-stimulation (4.38-fold) (*P* < 0.01) (**Figure 2C**). In addition, in comparison with the control group, a similar trend of significant up-regulation of *TroBcl2* was also found in the head kidney at time points from 6 h to 48 h after challenge, with a peak expression level (at 24 h post-stimulation) that was 3.17-fold greater than that in the control group (*P* < 0.01) (**Figure 2D**).

### Subcellular localization

**Figure 3:**
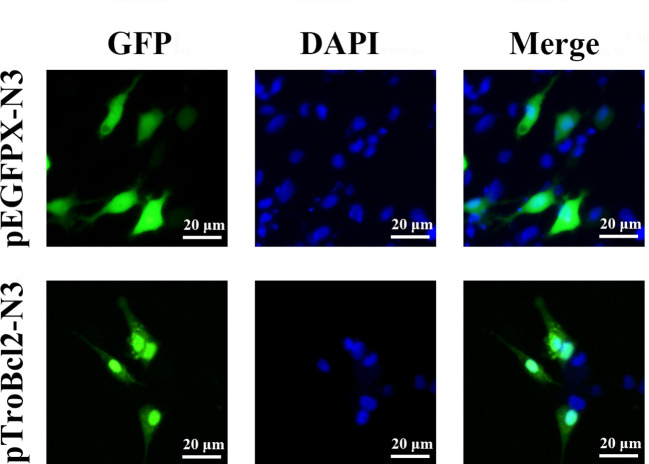
Subcellular distribution of TroBcl2. Golden pompano snout (GPS) cells were transiently transfected with plasmid pTroBcl2-N3 or pEGFPX-N3 for 48 h, and then examined using an inverted florescence microscope DMi8. DAPI staining shows the nuclei of cells.

To explore the subcellular localization of TroBcl2, the plasmid pTroBcl2-N3, which expresses both the TroBcl2 protein and GFP was transfected into GPS cells. As shown in **Figure 3**, the green florescence signals in cells transfected with pTroBcl2-N3 were distributed in both the cytoplasm and nucleus, indicating that TroBcl2 functions mainly in the cytoplasm but also in the nucleus. Not surprisingly, the green florescent signals in pEGFPX-N3-transfected cells were also present in the cytoplasm and nucleus. Moreover, the same distribution of TroBcl2 was shown in western blotting results (**Figure S2**).

### TroBcl2 inhibited apoptosis

**Figure 4:**
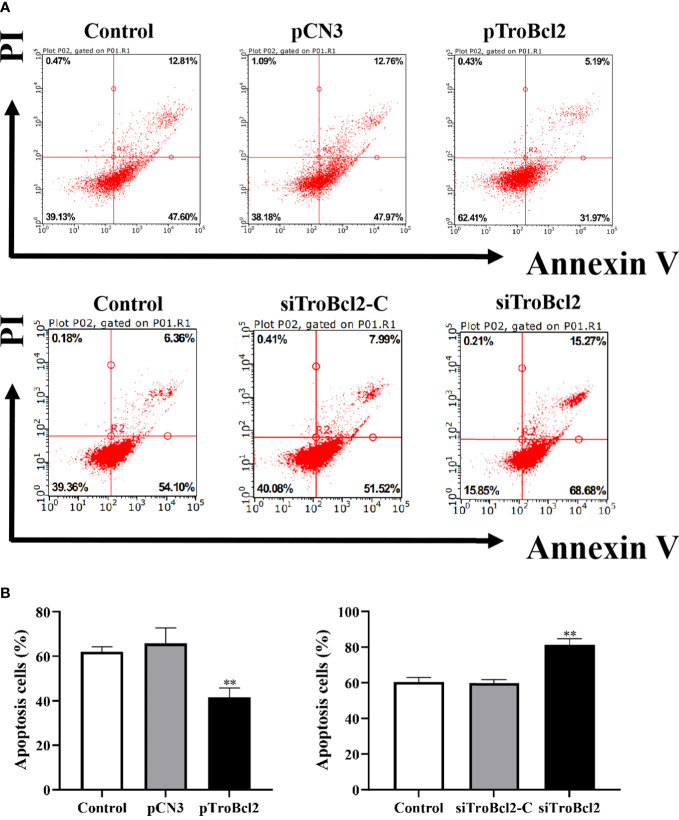
Effects of TroBcl2 overexpression and knockdown on cell apoptosis upon LPS stimulation. GPS cells transfected with pTroBcl2 or pCN3 were treated with LPS and the cell apoptosis was evaluated by flow cytometry **(A-1)**, and the bar chart represents the percentage of apoptotic cells **(B-1)**. Non-transfected cells treated with LPS were as the control. GPS cells transfected with siTroBcl2 or siTroBcl2-C were treated with LPS and the cell apoptosis was evaluated by flow cytometry **(A-2)**, and the bar chart represents the percentage of apoptotic cells **(B-2)**. Non-transfected cells treated with LPS were as the control. Data in **(A)** are one representative of three independent experiments, and **(B)** are presented as means ± SD (N = 3). N, experimental number. **P < 0.01.

Flow cytometry was used to assess the antiapoptotic property of TroBcl2. The overexpression and knockdown efficiencies of TroBcl2 in GPS cells were respectively detected firstly. Results showed that, after TroBcl2 overexpression in cells for 24 h, the mRNA expression level of *TroBcl2* was significantly increased by 527.22-fold relative to that of the control cells (*P* < 0.01) (**Figure S1A**). Similarly, the protein level of TroBcl2 was also successfully increased (**Figure S1C**). When TroBcl2 was knocked down in cells, the expression of *TroBcl2* mRNA in siTroBcl2-transfected cells was significantly decreased to 0.22-fold of that in control cells (**Figure S1B**). Similar results were also found in the protein levels of TroBcl2 post siTroBcl2 transfection (**Figure S1D**). Upon successfully overexpression and knockdown of TroBcl2 in cells, the cell apoptosis rates were then investigated by flow cytometry. As shown in **Figure 4A-1**, the apoptosis rate in pTroBcl2 transfected cells was 37.59%, which was lower than that in the control group (60.87%) and pCN3 group (61.82%). Correspondingly, the results in **Figure 4B-1** show that TroBcl2 overexpression significantly decreased the apoptosis rate compared with those in the pCN3 and control groups (*P* < 0.01). In addition, the apoptosis rate was not significantly different between the pCN3 and control groups (*P* > 0.05). However, the apoptosis rate of GPS cells in the siTroBcl2 group was higher (84.15%) compared with the siTroBcl2-C group (59.92%) and control group (60.64%) (**Figure 4A-2**), and the corresponding bar chart in **Figure 4B-2** confirmed the significant up-regulation of apoptosis rate of GPS cells when TroBcl2 was interfered (*P* < 0.01).

### TroBcl2 inhibited MMP loss and DNA fragmentation

**Figure 5:**
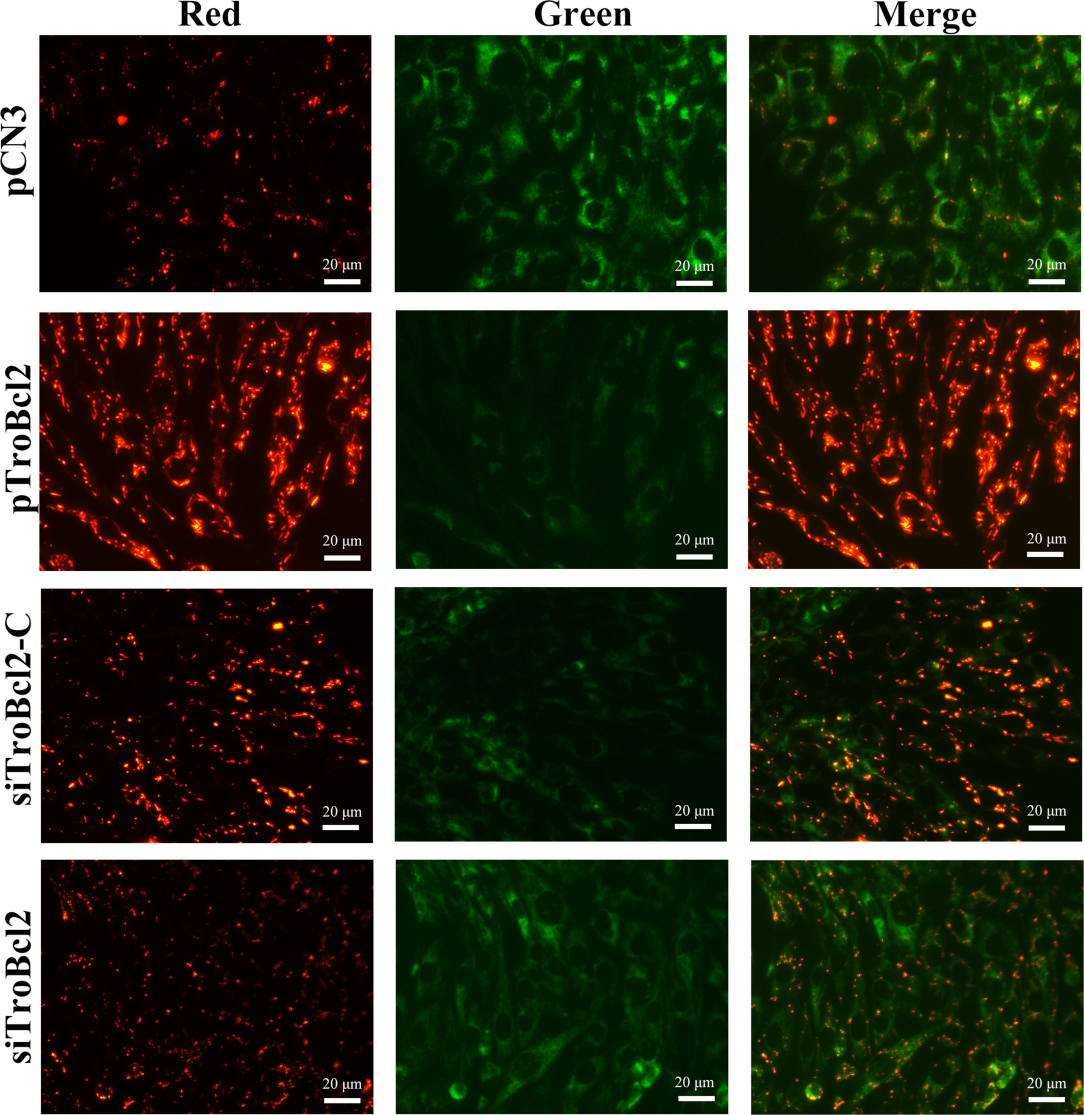
Effect of TroBcl2 overexpression and knockdown on mitochondrial membrane potential (MMP) upon LPS stimulation. GPS cells were transfected with pTroBcl2, pCN3, siTroBcl2, or siTroBcl2-C. Following 24 h transfection, cells were treated with LPS and the MMP change was measured by enhanced mitochondrial membrane potential assay kit with JC-1. The red and green fluorescences were observed by inverted fluorescence microscope DMi8.

To determine the potential reason that TroBcl2 inhibits apoptosis, we measured the change in the MMP, which is usually used as one of the indicators of early apoptosis (41). After LPS stimulation for 24 h, the marked green fluorescence signals as well as the faint red fluorescence signals were concentrated in control cells (pCN3 transfected), which showed that the MMP decreased significantly or even almost disappeared (**Figure 5**). It is obvious that the opposite effect occurred in TroBcl2-overexpressing cells, in which JC-1 dye was observed in mitochondria, with bright red fluorescence and weak green fluorescence (**Figure 5**). In contrast, compared with siTroBcl2-C group, more green fluorescence and less red fluorescence were presented in cells transfected with siTroBcl2, indicating TroBcl2 knockdown accelerated the loss of MMP (**Figure 5**).

**Figure 6:**
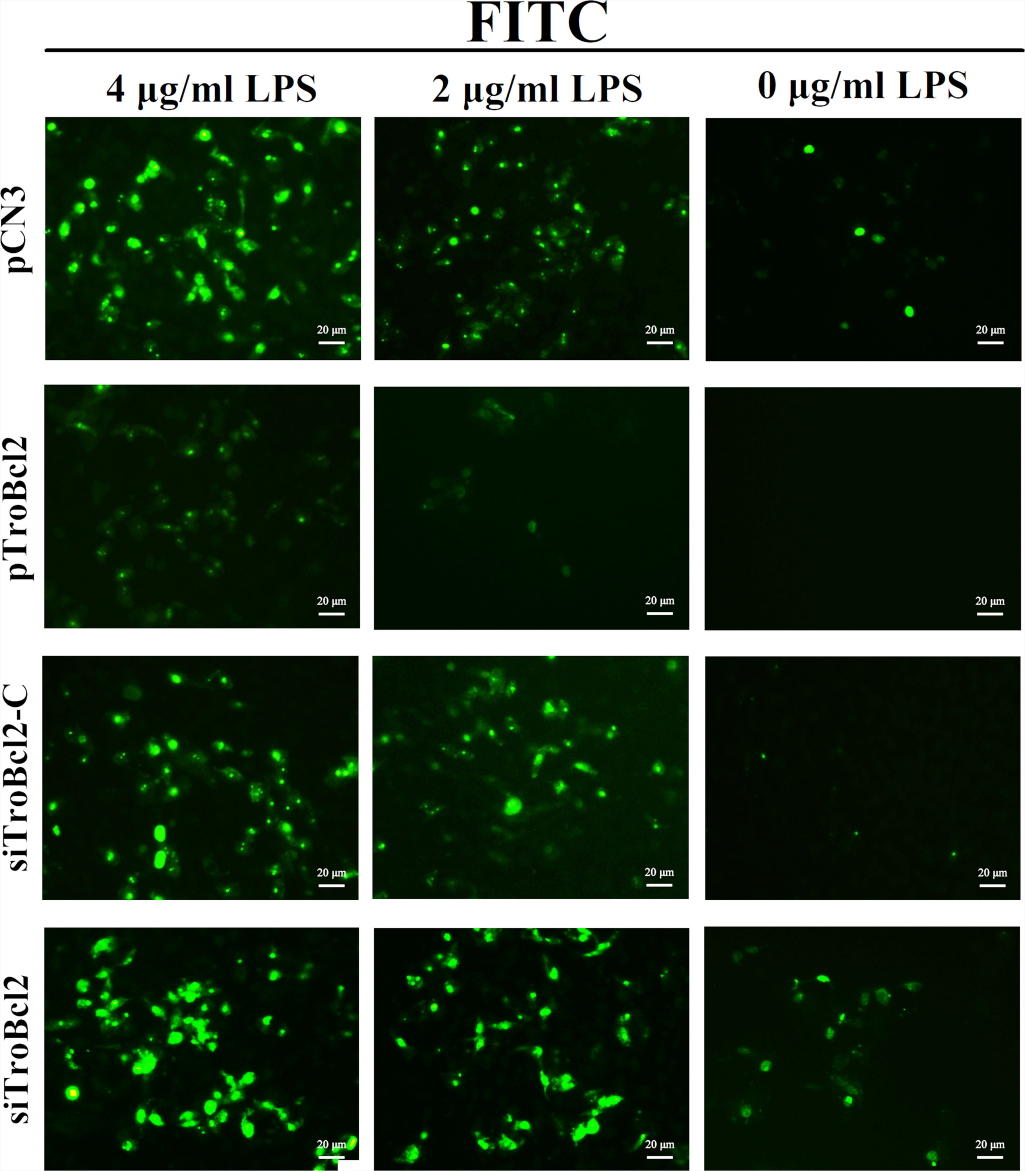
Effect of TroBcl2 overexpression and knockdown on LPS-induced apoptosis by TUNEL analysis. GPS cells were transfected with pTroBcl2, pCN3, siTroBcl2, or siTroBcl2-C. Following 24 h transfection, cells were treated with LPS and the apoptotic cells were measured by a TUNEL apoptosis assay kit. The green fluorescences were observed by inverted fluorescence microscope DMi8.

Furthermore, DNA fragmentation induced by endonucleases during apoptosis is another typical cellular apoptosis marker (42). Transfected cells were treated with different concentrations of LPS, and then apoptotic cells were detected by a TUNEL assay. As shown in **Figure 6A**, TUNEL-positive cells with bright green fluorescence were observed in the pCN3 group in a dose-dependent manner. In contrast, TroBcl2 exhibited a strong dose-dependent inhibitory effect on apoptosis, with fewer and weakly stained TUNEL-positive cells observed in this group (**Figure 6A**). In cells transfected with siTroBcl2, the percentage of TUNEL-positive cells were greater than that in cells transfected with siTroBcl2-C, suggesting that TroBcl2 knockdown increased DNA fragmentation (**Figure 6B**).

### TroBcl2 reduced cytochrome c release

**Figure 7:**
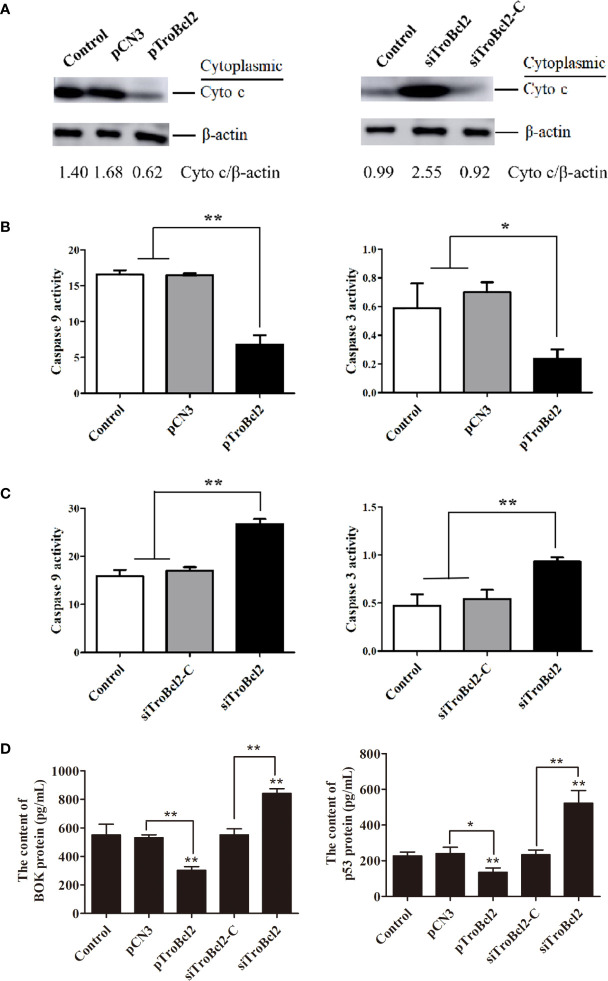
Effect of TroBcl2 overexpression and knockdown on the cytochrome c release into cytoplasm, caspase activities, and BOK and p53 protein levels following LPS stimulation. GPS cells transfected with pTroBcl2 and pCN3 or siTroBcl2 and siTroBcl2-Cfor 24 h were treated with LPS. Non-transfected cells treated with LPS were as the control. Then, the cytochrome c protein level in cytoplasm was detected by western blotting, and β-actin was used as the internal control **(A)**. The gray values of cytochrome c/β-actin are calculated by Image J and shown on the last line. The levels of caspase 3 and caspase 9 activation in cells were detected by the Caspase 3 and Caspase 9 Activity Assay Kits **(B, C)**. The protein levels of BOK and p53 were detected by ELISA assay **(D)**. Error bars display means ± SD (N = 3). N, experimental number. **P < 0.01, *P < 0.05.

The loss of MMP will lead to the permeability of the mitochondrial membrane and the release of cytochrome c from mitochondria. Given that TroBcl2 inhibited MMP loss, we wanted to know if it inhibited cytochrome c release. The cytochrome c protein in cytoplasm was detected by western blotting. Results showed that after LPS treatment, the cytochrome c protein could be detected in the control and pCN3 groups. However, the content of cytochrome c protein in the pTroBcl2 group was obviously reduced compared with the control and pCN3 groups, suggesting that the release of cytochrome c into the cytoplasm was inhibited by TroBcl2 (**Figure 7A**). In addition, TroBcl2 knockdown promoted cytochrome c release into cytoplasm as compared with cells in control and siTroBcl2-C groups (**Figure 7A**).

### TroBcl2 suppressed caspase 3 and caspase 9 activations

In order to verify whether the inhibitory effect of TroBcl2 on apoptosis is related to caspase pathway, we assayed the activities of caspase 3 and caspase 9. Interestingly, both of the activities of caspase-3 and caspase 9 in pTroBcl2-transfected cells were significantly decreased compared to that in the control and pCN3-transfected cells (**Figure 7B**). Corroborating each other, both of caspase-3 and caspase 9 showed significantly higher activities in siTroBcl2-transfected cells in comparison to that in the control and siTroBcl2-C-transfected cells (**Figure 7C**). These finding suggested that TroBcl2 suppressed caspase 3 and caspase 9 activations.

### Effect of TroBcl2 on the expression of apoptosis-related genes

**Figure 8:**
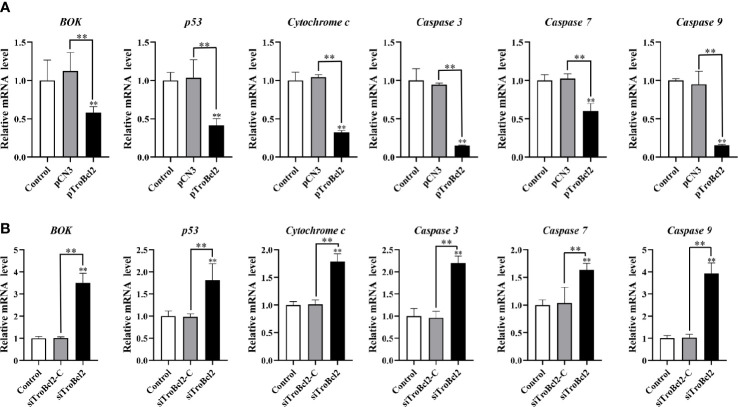
Effect of TroBcl2 overexpression and knockdown on the expression of apoptosis-related genes following LPS stimulation. qRT-PCR analysis of the expression levels of apoptosis-related genes in GPS cells transfected with pTroBcl2 and pCN3 **(A)** or siTroBcl2 and siTroBcl2-C **(B)** upon LPS treatment. Non-transfected cells treated with LPS were as the control. Error bars display means ± SD (N = 3). N, experimental number. **P < 0.01.

The mRNA levels of several apoptosis-related genes in cells overexpressed or interfered TroBcl2 were explored after LPS stimulation. **Figure 8A** shows the significant decreases in the *caspase 3* (0.15-fold), *caspase 9* (0.15-fold), *cytochrome c* (0.32-fold), *p53* (0.41-fold), *BOK* (0.58-fold), and *caspase 7* (0.60-fold) mRNA expression levels in pTroBcl2-overexpressing cells compared with control cells 24 h after LPS stimulation. However, the corresponding expression levels in pCN3-overexpressing cells were comparable to those in control cells. Correspondingly, both of the BOK and p53 protein levels were significantly decreased in pTroBcl2-overexpressing cells (**Figure 7D**).

Following LPS treatment, the mRNA expression of *cytochrome c*, *caspase 7*, *p53*, *caspase 3*, *BOK*, and *caspase 9* in cells transfected with siTroBcl2 was significantly enhanced by 1.79-fold, 1.63-fold, 1.81-fold, 2.20-fold, 3.50-fold, and 3.93-fold, respectively. In contrast, no significant differences in the mRNA expression levels of these apoptosis-related genes were found between cells transfected with siTroBcl2-C and the control non-transfected cells (**Figure 8B**). Similarly, both of the BOK and p53 protein levels were significantly increased in in cells transfected with siTroBcl2 (**Figure 7D**).

### TroBcl2 affects the expression of genes related to NF-κB signaling pathway and induces NF-κB promoter activity

**Figure 9:**
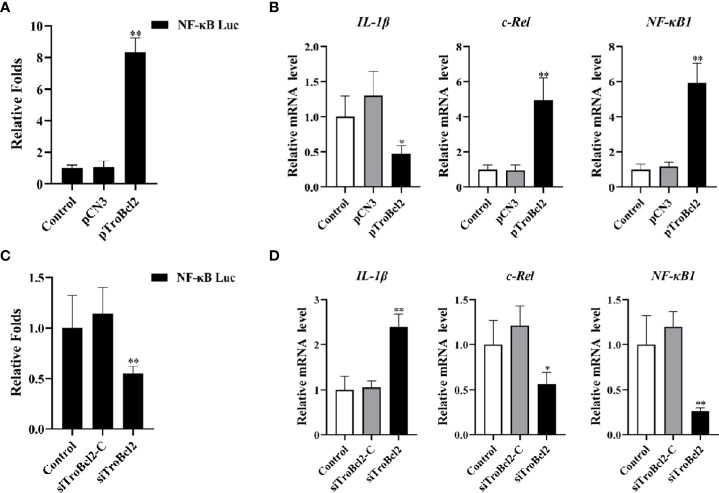
Effect of TroBcl2 overexpression and knockdown on the expression of NF-κB signaling pathway-related genes and NF-κB promoter activation following LPS stimulation. **(A)** GPS cells were co-transfected with NF-κB-pGL 4.32, pRL-CMV, pTroBcl2 (or pCN3), then treated with LPS. Non-transfected cells treated with LPS were as the control. The luciferase activity of the NF-κB promoter in the cells was detected. **(C)** GPS cells were co-transfected with NF-κB-pGL 4.32, pRL-CMV, siTroBcl2 (or siTroBcl2-C), then treated with LPS. Non-transfected cells treated with LPS were as the control. The luciferase activity of the NF-κB promoter in the cells was detected. qRT-PCR analysis of the expression levels of *NF-κB1*, *c-Rel*, and *IL-1β* in GPS cells transfected with pTroBcl2 and pCN3 **(B)** or siTroBcl2 and siTroBcl2-C **(D)** upon LPS treatment. Non-transfected cells treated with LPS were as the control. Error bars display means ± SD (N = 3). N, experimental number. **P < 0.01.

Our results suggested that the transcript levels of *NF-κB1* and *c-Rel* were significantly increased (5.92-fold and 4.97-fold, respectively) in response to TroBcl2 overexpression in the presence of LPS (**Figure 9B**). However, the transcript level of the inflammatory cytokine *IL-1β* was markedly reduced (0.47-fold) when TroBcl2 was overexpressed after stimulation with LPS. In contrast, the trends in the levels of the *NF-κB1*, *c-Rel*, and *IL-1β* transcripts were reversed in cells in which TroBcl2 was knocked down (**Figure 9D**). Based on these findings, we further investigated whether TroBcl2 induces the activation of NF-κB. From the results of the luciferase reporter assay, we clearly observed the induction of NF-κB promoter activity in TroBcl2-transfected GPS cells (**Figure 9A**). The corroborating evidence showed that the luciferase activity of NF-κB in cells with TroBcl2 interference was significantly reduced to 0.55-fold of that in control cells (**Figure 9C**). In addition, transfection of pCN3 or siTroBcl2-C failed to activate NF-κB transcription.

## Discussion

Bcl-2 plays an important role in the mammalian apoptotic process (31). However, there are few reports on the functions of Bcl-2 in fish. Here, Bcl-2 of *T. ovatus* was cloned and characterized, and its effects on the apoptotic process were investigated. Our findings revealed that TroBcl2 showed conserved anti-apoptotic activity in GPS cells by inhibiting MMP loss and DNA fragmentation upon LPS stimulation. Moreover, its anti-apoptotic activity might be associated with decreased cytochrome c release, inhibition of the p53 pathway and caspase signaling cascade, and activation of the NF-κB.

As reported, BH domains are typical structural features of Bcl-2 family members, and are essential for the function of Bcl-2 family members (43). In this study, sequence analysis showed that TroBcl2 has four conserved BH domains, especially BH4, indicating that TroBcl2 is an anti-apoptotic protein. The conserved BH1 and BH2 domains in TroBcl2 might provide a structural basis for the formation of heterodimers between TroBcl2 and pro-apoptotic proteins in *T. ovatus*, although this possibility needs further validation. Moreover, a signature “NWGR” motif located in the BH1 domain, which is an essential site for Bcl-2/Bax heterodimerization and Bcl-2/Bcl-2 homodimerization and has been confirmed to be necessary for the anti-apoptotic function of Bcl-2 family members, was found in TroBcl2 (44–46). Similarly, Bcl-2 in mammals (47), *D. rerio* (35), *E. coioides* (4), *C. striatus* (2) and *Pinctada fucata martensii* (48) contains four BH domains (BH1, BH2, BH3, and BH4). Surprisingly, the structural composition of Bcl-2 appears to be somewhat different in some invertebrate aquatic animals. For example, Bcl-2 of *Scylla paramamosain* (49), *Apostichopus japonicus* (50), and *Portunus trituberculatus* (15) contains only three BH domains (i.e., BH1, BH2 and BH3), indicating that BH4 may not be important in these invertebrate aquatic animals. Given that BH4 differs among species of different phylogenies, its function needs to be further investigated.

Apoptosis is an important part of the normal regeneration process of various tissues and cells (2, 3). As an important regulator, Bcl-2 is widely distributed in important immune and hematopoietic organs of vertebrates and can regulate apoptosis in a tissue-specific manner (51, 52). In this study, TroBcl2 mRNA was detected in the eleven tested tissues and was highly expressed in the brain, spleen, head kidney, and liver. Similar observations have been reported for *C. striatus* Bcl-2, which had a distribution pattern from high to low in spleen, kidney, head kidney, blood and liver (2). Because TroBcl2 is highly expressed in immune-related tissues and the vital role of Bcl-2 in immune defense against bacterial pathogens was revealed by previous reports, we then explored the changes in TroBcl2 expression in the spleen and head kidney in response to LPS. Our results suggested that TroBcl2 transcription in the spleen and head kidney were significantly up-regulated at 9 h, 12 h and 24 h post challenge with LPS, peaking at 12 h and then decreasing. After stimulation by LPS, a similar increasing trend in Bcl-2 expression was found in *C. striatus* (2), *Chlamys farreri* (53), and *P. fucata martensii* (48). Therefore, TroBcl2 might also be involved in the host immune response to bacteria.

The anti-apoptotic activity of Bcl-2 has been extensively demonstrated in mammals (28, 29, 54). Suppression of the cell death process by Bcl-2 mainly depends on inhibiting the expression of pro-apoptotic proteins (such as Bax and Bak) to maintain the integrity of mitochondrial outer membrane (31). However, whether Bcl-2 has a conserved anti-apoptotic function in *T. ovatus* remains unknown. Subcellular localization analysis is an indispensable technical means to study gene function, which can provide research directions for understanding the mechanisms of gene action. In the present study, we explored the subcellular localization of TroBcl2 in GPS cells and found that TroBcl2 was distributed in the cytoplasm and nucleus. In previous evidences, Bcl-2 was mostly described to be present in multiple subcellular localizations, i.e., in nuclear outer membrane, endoplasmic reticulum membrane, and mitochondrial membranes. Even so, several studies have confirmed that Bcl-2 can be localized not only in the cytoplasm but also in the nucleus (4, 55, 56). For example, Hoetelmans et al. (56) found that transfection of human Bcl-2 into rat cells resulted in cytoplasmic and nuclear Bcl-2, which was in line with increasing evidences that the role of the Bcl-2 family of proteins should be extended to activities inside the nuclear compartment. In teleost, Bcl-2 in *E. coioides* was also found to be localized in both nucleus and cytoplasm (4). There is no doubt that cytoplasmic localization of Bcl-2 is closely related to apoptosis, but the function of Bcl-2 in the nucleus remains to be further studied. The localization of TroBcl2 provides favorable evidence that TroBcl2 is a member of the anti-apoptotic Bcl-2 subfamily and suggests that it may exert the conserved function in cytoplasm. As expected, flow cytometric analysis showed that overexpression of TroBcl2 significantly decreased the apoptosis rates of GPS cells following LPS-mediated induction of apoptosis, while the apoptosis was promoted when TroBcl2 was interfered. Collectively, these results suggest that TroBcl2 possesses anti-apoptotic function, similar to Bcl-2 in mammals.

There is widespread agreement that in mammals, mitochondria play a central role in the process of apoptosis and that Bcl-2 performs its anti-apoptotic function through the mitochondrial pathway (31, 57). To further investigate the potential mechanism underlying the anti-apoptotic activity of TroBcl2, we checked the typical biochemical changes involved in apoptosis, including the MMP change and DNA fragmentation. A decrease in the MMP is considered to be an important characteristic of apoptotic cells and is the earliest event in the process of apoptosis (occurring prior to nuclear morphological changes and phosphatidylserine eversion). The cells enter an irreversible process of apoptosis when MMP is lost (58). Moreover, when cells undergo apoptosis, endonuclease enzymes that cleave the genomic DNA between nucleosomes are activated, resulting in DNA fragmentation. Therefore, the status of apoptosis can be indicated by detecting DNA fragmentation (59). In our study, TroBcl2 significantly inhibited MMP loss and DNA fragmentation upon LPS-induced apoptosis, suggesting that TroBcl2 may block apoptosis in fish cells through the mitochondrial pathway. Consistent with our findings, overexpression of Bcl-2 in grouper fin cells reduced DNA fragmentation and MMP loss induced by the serine/threonine (ST) kinase of SGIV (57). In addition, a growing number of studies have provided evidence that overexpression of Bcl-2 can protect many different cell lines from apoptosis induced by a wide variety of stimuli through the mitochondrial pathway (27, 29, 60).

The process of apoptosis mediated through the mitochondrial pathway is regulated by a series of related molecules in the cell. Upon exposure to apoptotic stimuli, pro-apoptotic Bcl-2 subfamily proteins (such as Bax and Bak) located in the cytosol translocate to the outer mitochondrial membrane and change the permeability of the outer mitochondrial membrane, leading to the increased permeability of the outer mitochondrial membrane (61). What happens next is the release of pro-apoptotic substances such as cytochrome c from mitochondria into the cytoplasm, which then activates caspase 9 and caspase 3, leading to cell apoptosis (62). However, all these events can be blocked by Bcl-2, which can form heterodimers by binding to pro-apoptotic proteins to prevent pro-apoptotic proteins from destroying the integrity of the outer mitochondrial membrane and subsequently inducing apoptosis (63). In the present study, we also found that TroBcl2 could prevent the release of cytochrome c into the cytoplasm, which may echo the finding that TroBcl2 inhibited the loss of MMP. Previous studies have shown that cytochrome c may be necessary for the activation of caspase 3 and that Bcl-2 blocks the mitochondrial release of cytochrome c, which is now known to be involved in the activation of caspase 9 (64). We revealed that TroBcl2 indeed reduced the activities both of caspase 3 and caspase 9, indicating the anti-apoptotic activity of TroBcl2 might be achieved *via* a caspase-dependent pathway. Furthermore, we verified the effect of TroBcl2 on the expression of apoptosis-related genes in the mitochondrial pathway. Not surprisingly, overexpression of TroBcl2 significantly downregulated the expression of *cytochrome c*, *caspase 9*, *caspase 7*, and *caspase 3* in LPS-treated cells. In contrast, these expression levels were significantly increased when TroBcl2 was knocked down, confirming that TroBcl2 might exert the anti-apoptotic effects by inhibiting cytochrome c release and regulating the caspase signaling cascade. In addition, we found that *p53*, an apoptosis-related gene that can induce apoptosis in cells with DNA damage, was negatively regulated by TroBcl2. Previous studies have reported that p53 signaling is a crucial pathway that participates in the regulation of the apoptotic signaling cascade, which has been shown to be a direct transcriptional activator of Bax/Bak (9). (65) revealed that the protein level of p53 increased during Chinese giant salamander iridovirus (GSIV)-induced apoptosis in *Andrias davidianus* and that the anti-apoptotic molecule AdBcl-xL inhibited the anti-apoptotic effect of p53. Therefore, our results suggest that the anti-apoptotic activity of TroBcl2 may be related to inhibition of p53 activation. It is noteworthy in this study that the expression of BOK, a pro-apoptotic molecule, was significantly inhibited in cells overexpressing TroBcl2 upon LPS stimulation, whereas conversely *BOK* expression was significantly increased when TroBcl2 was suppressed. However, Hsu et al. (62) found that rat Bok could not bind to anti-apoptotic proteins (Bcl-2, Bcl-xL, or Bcl-w), except for Mcl-1, BHRF1, and Bfl-1, unlike other pro-apoptotic proteins (such as Bax, Bak, and Bik) that bind directly to all anti-apoptotic proteins. Further verification showed that, co-transfection of BOK and Mcl or BHRF1 in Chinese hamster ovary (CHO) cells inhibited BOK-induced apoptosis, but co-expression of BOK with Bcl-2 was ineffective in blocking the action of BOK. Inohara et al. (66) also reported that mouse Bok (also known as Mtd) promotes apoptosis in the absence of direct interactions with survival-promoting Bcl-2 and Bcl-X_L_. In a previous study, mammalian BOK appeared to be constitutively active and unresponsive to the antagonistic effects of the anti-apoptotic Bcl-2 proteins and was stabilized to induce MOMP and apoptosis independent of other Bcl-2 proteins (47). As far as available reports are concerned, the current connection between Bok and Bcl-2 remains unknown. Hence, based on the interesting finding that TroBcl2 negatively regulated *BOK* transcription in this study, the role of BOK in the anti-apoptotic function of TroBcl2 needs to be further explored in the future.

To our knowledge, NF-κB is an important nuclear transcription factor that is involved in cell survival, apoptosis and proliferation and shows a marked ability to inhibit apoptosis and the caspase cascade (67–69). In our study, TroBcl2 overexpression increased the transcript levels of *NF-κB1* and *c-Rel*, which are key members of the NF-κB transcription factor family, suggesting that the protective effect of TroBcl2 against LPS-mediated apoptosis might be related to the NF-κB signaling pathway. Moreover, IL-1β, a key pro-inflammatory cytokine, is involved in a variety of cellular activities (including proliferation, differentiation, and apoptosis), and its induction can be regulated by NF-κB (70). In a previous study, Bcl-2 overexpression decreased the levels of LPS-induced proinflammatory cytokines (such as *IL-6* and *TNF-α*) to protect cortical neural stem cells (71). Similarly, decreased expression of *IL-1β* in TroBcl2-overexpressing cells in this study might suggest that TroBcl2 acts as an anti-inflammatory regulator. To verify our hypothesis, we investigated the effect of TroBcl2 on the transcription of NF-κB. Our data confirmed that TroBcl2 indeed activated the transcription of NF-κB. Consistent with this finding, de Moissac et al. (72) provided the evidence that Bcl-2 can increase the transcription of NF-κB through IκBα degradation. Thus, our results revealed that the transcriptional regulation of NF-κB by Bcl-2 in *T. ovatus* was similar to that in mammals and that TroBcl2 executed its anti-apoptotic activity in a manner possibly dependent on NF-κB activation.

## Conclusion

In summary, a Bcl-2 gene from *T. ovatus*, named as TroBcl2, was identified and characterized for the first time. Moreover, TroBcl2 was widely expressed in various tissues and upregulated in immune related tissues in response to LPS stimulation. TroBcl2 exhibited the anti-apoptotic activity and inhibited the apoptotic process *via* preventing the MMP loss and DNA fragmentation induced by LPS. More importantly, the anti-apoptotic activity of TroBcl2 was achieved by preventing cytochrome c release, inhibiting the p53 pathway and caspase signaling cascade, and activating NF-κB transcription.

## Data availability statement

The raw data supporting the conclusions of this article will be made available by the authors, without undue reservation.

## Ethics statement

The animal study was reviewed and approved by The Committee and Laboratory Animal Department of Hainan University.

## Author contributions

ZC contributed to the conception, design, data acquisition, and drafting of the article. XY and TL contributed to data acquisition. ZL, YZ, and PL contributed to writing—review and editing. YS contributed to project administration and funding acquisition. All authors contributed to the article and approved the submitted version.
