# Supplementary material for: Molecular characterization and expression analysis of B-cell lymphoma-2 in Trachinotus ovatus and its role in apoptotic process
Source: Front Immunol. 2023 Mar 16;14:1129800. doi: 10.3389/fimmu.2023.1129800 (PMC10063160; doi:10.3389/fimmu.2023.1129800)
Supplement: Supplementary file 1 [file DataSheet_1.docx]

**SUPPLEMENTARY DATA**

**TABLE S1** Primers used in this study

| Primer name | Primer sequences (5’-3’) |  |
| --- | --- | --- |
| TroBcl2-F | GATATCGCCACCATGGCGAGCGAGTGTAATCGC | TroBcl2 ORF cloning |
| TroBcl2-R | GATATCCTTCTGTGTAAGGTACGCTCCGATG |  |
| TroBcl2-RT-F | CGAGCGAGTGTAATCGCAACA | qPCR |
| TroBcl2-RT-R | GCCATTATTAGCAGCGTCTTCATC |  |
| M13-F1 | CGCCAGGGTTTTCCCAGTCACGAC | Detection of TroBcl2 |
| M13-R1 | AGCGGATAACAATTTCACACAGGA |  |
| CN-F1 | CTTGCGTTTCTGATAGGCACCTA | Detection of pTroBcl2 |
| CN-R1 | TGCGGGCCTCTTCGCTATT |  |
| His-R | GTGGTGGTGGTGGTGGTG |  |
| B2M-F | AAGTCAGTCCACCCAAGGTTCA | qPCR |
| B2M-R | GGGATTTCCATTCCGTTCTTCATG |  |
| BOK-F | CGACCGTTCGTTGACTGAGAA | qPCR |
| BOK-R | CGCCGCATTTGAGGGAG |  |
| Cytochrome c-F | GGGTCTGTTCGGACGCAAGA | qPCR |
| Cytochrome c-R | GTTCTCCAGGTACTCCATCAAGGT |  |
| Caspase 3-F | AGCTGACTTCCTCTACGCCTTCT | qPCR |
| Caspase 3-R | CTACTGCCACCTTGTGGTTCACT |  |
| Caspase 7-F | AGCTCGTGTTTCGCCTGTATCCT | qPCR |
| Caspase 7-R | CCGACAAGCCTGGTGAAGTAA |  |
| Caspase 9-F | ACATAAGACCAAGGCCACAAGG | qPCR |
| Caspase 9-R | CTCGCTCTGAGGTTCAAAGTCC |  |
| p53-F | TCCTGGCTGAAGGATTTGATG | qPCR |
| p53-R | GAAGTGACGGACTTGGCTGTG |  |
| IL-1β-F | GGAGACTGTGGAGGACAAGAGC | qPCR |
| IL-1β-R | GCGGGCAGACATGAAGGTG |  |
| NF-κB1-F | CTGCGACAAAGTCCAGAAAGATG | qPCR |
| NF-κB1-R | CTGCGACAAAGTCCAGAAAGATG |  |
| c-Rel-F | TGGGCTCTTTCCCCTTCCTA | qPCR |
| c-Rel-R | ACGAGGAAGCAAGTTGGTGT |  |
| siTroBcl2-P1 | GGATCCTAATACGACTCACTATAGCGAGTGTAATCGCAACAT | Synthesizing siTroBcl2 |
| siTroBcl2-P2 | AAATGTTGCGATTACACTCGCTATAGTGAGTCGTATTAGGATCC |  |
| siTroBcl2-P3 | GGATCCTAATACGACTCACTATAATGTTGCGATTACACTCGC |  |
| siTroBcl2-P4 | AAGCGAGTGTAATCGCAACATTATAGTGAGTCGTATTAGGATCC |  |
| siTroBcl2-C-P1 | GGATCCTAATACGACTCACTATAGCCACAACGTCTATATCAT | Synthesizing siTroBcl2-C |
| siTroBcl2-C-P2 | AAATGATATAGACGTTGTGGCTATAGTGAGTCGTATTAGGATCC |  |
| siTroBcl2-C-P3 | GGATCCTAATACGACTCACTATAATGATATAGACGTTGTGGC |  |
| siTroBcl2-C-P4 | AAGCCACAACGTCTATATCATTATAGTGAGTCGTATTAGGATCC |  |

**TABLE S2** The amino acid sequence similarities of TroBcl2 with Bcl-2 from other species

| **Species** | **GenBank Accession number** | **Identities (%)** |
| --- | --- | --- |
| *Trachinotus ovatus* | OP784574 | 100 |
| *Toxotes jaculatrix* | XP_040910734.1 | 95.18 |
| *Seriola lalandi dorsalis* | XP_023282977.1 | 94.30 |
| *Amphiprion ocellaris* | XP_023143231.1 | 93.86 |
| *Larimichthys crocea* | XP_010728299.1 | 92.98 |
| *Mastacembelus armatus* | XP_026168664.1 | 90.79 |
| *Myripristis murdjan* | XP_029929517.1 | 87.72 |
| *Salmo salar* | XP_014026897.2 | 76.44 |
| *Oncorhynchus mykiss* | ASW34029.1 | 76.00 |
| *Danio rerio* | AAW21970.1 | 56.44 |
| *Homo sapiens* | API71152.1 | 50.28 |
| *Mus musculus* | AAH95964.1 | 49.77 |


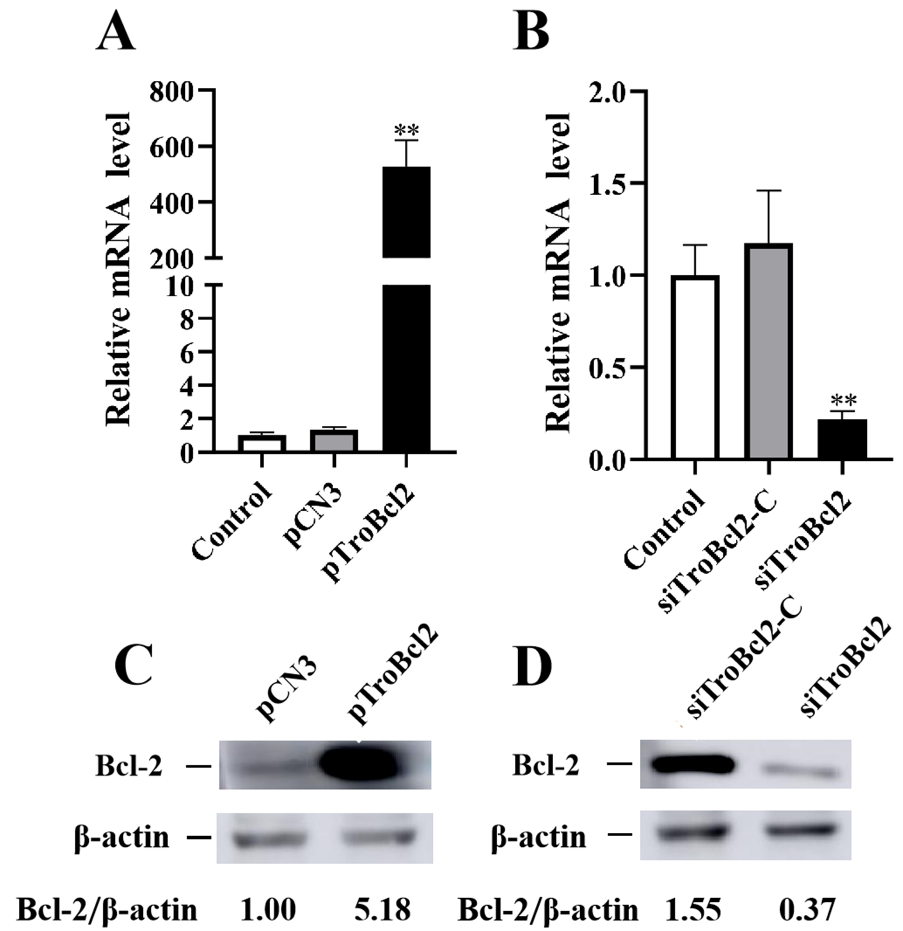


**FIGURE S1** The overexpression or knockdown efficiencies of TroBcl2 in GPS cells. (A) qRT-PCR analysis of the overexpression efficiency of TroBcl2 mRNA. (B) qRT-PCR analysis of the knockdown efficiency of TroBcl2 mRNA. Error bars display means ± SD (N = 3). N, experimental number. ***P* < 0.01. (C) Western blotting analysis of the overexpression efficiency of TroBcl2 protein. (D) Western blotting analysis of the knockdown efficiency of TroBcl2 protein. The gray values of Bcl-2/β-actin are calculated by Image J and shown on the last line.


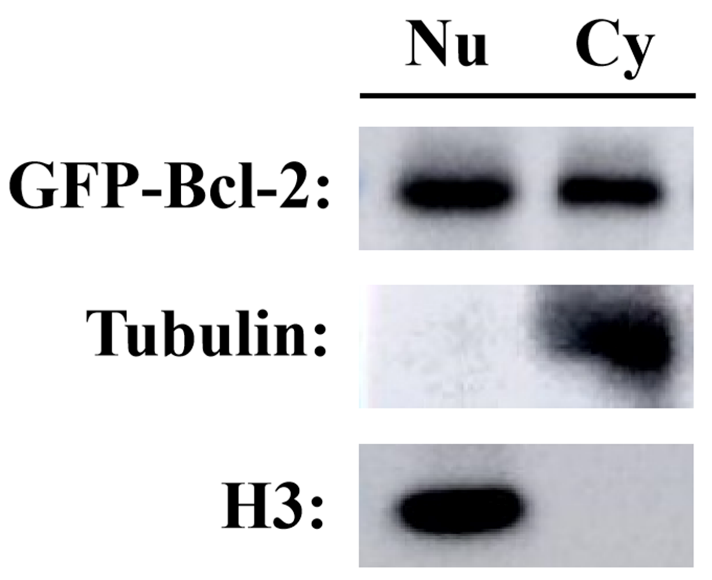


**FIGURE S2** The TroBcl2 subcellular localization. The nuclear and cytoplasmic protein were extracted from the cells transfected by pTroBcl2-N3. Then, the TroBcl2 protein in nucleus and cytoplasm were detected by western blotting with mouse anti-TroBcl2 polyclonal antibody. Nu means nucleus and Cy means cytoplasm. Tubulin, a marker protein in the cytoplasm; H3, a marker protein in the nucleus.
